# *Nod2* is required for antigen-specific humoral responses against antigens orally delivered using a recombinant *Lactobacillus* vaccine platform

**DOI:** 10.1371/journal.pone.0196950

**Published:** 2018-05-07

**Authors:** Sara A. Bumgardner, Lin Zhang, Alora S. LaVoy, Barbara Andre, Chad B. Frank, Akinobu Kajikawa, Todd R. Klaenhammer, Gregg A. Dean

**Affiliations:** 1 Center for Comparative Medicine and Translational Research, College of Veterinary Medicine, North Carolina State University, Raleigh, North Carolina, United States of America; 2 Department of Microbiology, Immunology, and Pathology, College of Veterinary Medicine and Biomedical Sciences, Colorado State University, Fort Collins, Colorado, United States of America; 3 Department of Applied Biology and Chemistry, Tokyo University of Agriculture, Setagaya, Tokyo, Japan; 4 Department of Food, Bioprocessing, & Nutrition Sciences, North Carolina State University, Raleigh, North Carolina, United States of America; Instituto Butantan, BRAZIL

## Abstract

Safe and efficacious orally-delivered mucosal vaccine platforms are desperately needed to combat the plethora of mucosally transmitted pathogens. *Lactobacillus* spp. have emerged as attractive candidates to meet this need and are known to activate the host innate immune response in a species- and strain-specific manner. For selected bacterial isolates and mutants, we investigated the role of key innate immune pathways required for induction of innate and subsequent adaptive immune responses. Co-culture of murine macrophages with *L*. *gasseri* (strain NCK1785), *L*. *acidophilus* (strain NCFM), or NCFM-derived mutants—NCK2025 and NCK2031—elicited an M2b-like phenotype associated with TH2 skewing and immune regulatory function. For NCFM, this M2b phenotype was dependent on expression of lipoteichoic acid and S layer proteins. Through the use of macrophage genetic knockouts, we identified Toll-like receptor 2 (TLR2), the cytosolic nucleotide-binding oligomerization domain containing 2 (NOD2) receptor, and the inflammasome-associated caspase-1 as contributors to macrophage activation, with NOD2 cooperating with caspase-1 to induce inflammasome derived interleukin (IL)-1β in a pyroptosis-independent fashion. Finally, utilizing an NCFM-based mucosal vaccine platform with surface expression of human immunodeficiency virus type 1 (HIV-1) Gag or membrane proximal external region (MPER), we demonstrated that NOD2 signaling is required for antigen-specific mucosal and systemic humoral responses. We show that lactobacilli differentially utilize innate immune pathways and highlight NOD2 as a key mediator of macrophage function and antigen-specific humoral responses to a *Lactobacillus acidophilus* mucosal vaccine platform.

## Introduction

Induction of mucosal immunity by direct vaccination is an attractive strategy to combat pathogens that are transmitted at mucosal surfaces. Mucosal vaccination is believed to be more effective than parenteral vaccination at inducing mucosal immunity. Nevertheless, the majority of vaccines are parentally delivered due to the lack of robust mucosal vaccine platforms (reviewed in [1, 2]).

Proof-of-principle studies have demonstrated that lactobacilli-based vaccine platforms are promising candidates for mucosal vaccination against a variety of pathogens including influenza, anthrax, severe acute respiratory syndrome (SARS), and human immunodeficiency virus (HIV) [3–6]. Lactobacilli are an attractive vaccine vehicle because they persist in the gastrointestinal tract, adhere to epithelial cells, and modulate the immune response through pattern recognition receptors (PRR) (reviewed in [7–10]). Lactobacilli are generally regarded as safe by the United States Food and Drug Administration and thus have a significant advantage over pathogen-based platforms. The challenge is to maintain the inherent safety of lactobacilli while maximizing immunogenicity against relevant pathogen epitopes.

Structural variations among strains of lactobacilli influence the type and strength of innate and adaptive immune responses elicited by the bacteria, but the mechanisms are incompletely understood [8, 11, 12]. We and others have shown that immune cell PRRs are activated by several cell wall components of lactobacilli [13–16]. These include nucleotide-binding oligomerization domain containing 2 (NOD2) activation by the peptidoglycan (PGN) component muramyl dipeptide (MDP), Toll-like receptor 2 (TLR2) and TLR2/6 activation by lipoproteins and lipoteichoic acids (LTA), and activation of C-type lectin receptors (CLR) by bacterial surface layer proteins (Slp) (reviewed in [10]). Lactobacilli have also been shown to activate the caspase-1 dependent inflammasome, resulting in the production of active IL-1β [7, 17].

To optimize a lactobacillus vaccine platform and elicit an efficacious mucosal immune response, a mechanistic understanding of PRR activation by candidate species, strains, and mutants is necessary. To that end, we investigated the immunomodulatory capabilities of *L*. *gasseri*, *L*. *acidophilus* (strain NCFM), and NCFM mutant strains. We employed bone marrow-derived macrophages (BMDM) from wild-type and specific PRR-deficient mice to examine the role of TLR2, NOD2, and caspase-1 stimulation on macrophage activation and cytokine production. In a novel observation, we reveal NOD2 signaling is essential for antigen-specific mucosal IgA responses *in vivo*.

## Materials and methods

### Ethics statement

This study was carried out in strict accordance with the Guide for the Care and Use of Laboratory Animals of the National Institutes of Health and the Association for the Assessment of Laboratory Animal Care standards and with approval from the Institutional Animal Care and Use Committee of North Carolina State University (protocol number 11-049-B and 09-127-B) and Colorado State University (protocol number 17-7495A), where applicable. Animal welfare and health was monitored daily. All animals were humanely euthanized at study endpoint via carbon dioxide inhalation, confirmed with physical examination, and ensured with secondary pneumothorax; and every effort was made to minimize suffering.

### Mice

Wild type BALB/cJ and C57BL/6J mice along with mutant knockout mice B6.129-Tlr2^tm1Kir^/J, B6.129S1-Nod2^tm1Flv^/J, and B6.129S1-Tlr5^tm1Flv^/J were purchased from The Jackson Laboratory (Bar Harbor, ME, USA). *Caspase1-/-* on the C57BL/6 background were generously gifted from Dr. J. P. Ting (UNC-Chapel Hill) [18]. *Nod2*-/- mice on the BALB/c background were generously provided by Dr. Holly Rosenzweig (Oregon Health & Science University), bred at CSU, and *Nod2-/-* genotype was confirmed [19].

All animals were age matched (8–12 weeks), maintained in specific pathogen free conditions, individually tracked and monitored daily for clinical signs of stress or illness, including but not limited to changes in skin and hair, eyes and mucous membranes, respiratory system, circulatory system, central nervous system, salivation, diarrhea, or lethargy. Upon arrival, animals were housed socially (2–5) in commercially available, individually ventilated caging systems with a 12 h light/12 h dark cycle. Animals were provided *ad libitum* commercial irradiated rodent chow (Teklad Global) and tap water filtered via reverse osmosis in autoclaved water bottles; all bedding and enrichment materials was autoclaved prior to use and changed regularly.

### Lactobacilli preparation

Lactobacilli were grown in either MRS broth (BD, Franklin Lakes, NJ) or MRS with 5 μg/mL erythromycin under anaerobic conditions to log phase when used live or to static phase when used as UV-killed (1h exposure). For phagocytosis assays, static phase lactobacilli were washed twice with phosphate buffered saline (PBS) then labeled with 0 or 50μM CellTrace Violet or 50μM 5-(and-6)-carboxyfluorescein diacetate, succinimidyl ester (CFSE; both from Life Technologies, Grand Island, NY) for flow cytometry or fluorescence microscopy. Labeled bacteria were washed twice with PBS containing 10% FBS and suspended in macrophage medium (see below). Lactobacilli CFU and UV-killing were verified by plating on MRS agar.

All *Lactobacillus* strains used in this study are listed in Table 1. Recombinant strains were engineered to express HIV-1 Gag or MPER (NEQELLELDKWASLWN) on the cell surface as previously described [6, 20]. For intragastric dosing, bacteria were washed twice in sterile PBS and suspended in 0.1 M sodium bicarbonate with 20 mg/mL soybean trypsin inhibitor (Sigma-Aldrich, St. Louis, MO) to protect surface antigens [6, 21].

**Table 1:** Bacterial strains used.

Strain	Genotype or Characteristic(s)	Reference(s)
*L*.*acidophilus* strains		
NCFM	Human intestinal isolate	[22]
NCK2025	NCFM with deletion of phosphoglycerol transferase gene (LBA0447): LTA^-^	[23]
NCK2031	NCFMΔ*upp* with sequential deletion of *slpB* (LBA0175) and *slpX* (LBA0512): SlpB^-^SlpX^-^SlpA^+^	[24, 25]
NCK1895	NCFM containing pTRK882 with *pgm* operon regulatory element; Em^r^	[6, 26]
NCK2166	NCFM containing pTRK1037, derivative of pTRK882; cell surface expression of HIV Gag; Em^r^	[6]
GAD31	NCK2208 (Δ*upp*, slpA-MPER) with pTRK882; HIV MPER (NEQELLELDKWASLWN) integrated into SlpA; Em^r^	[20]
*L*.*gasseri* strains		
NCK1785	ATCC33323 harboring Em^r^ pTRK563 plasmid	[16]

### Lysozyme digestion

2.8 × 10^8^ CFU/mL of live or UV-killed lactobacilli were suspended with or without 100μg/mL lysozyme from chicken egg white (L7651, Sigma-Aldrich) and incubated aerobically at 37 °C. At 0, 1, and 2 h, samples were frozen at—80 °C for subsequent hNOD2 stimulation assays or used immediately for OD_600_ evaluation. To assess degradation of lactobacilli cell wall, duplicate bacterial digests were heated at 94 °C for 5 minutes to inactivate lysozyme, suspended in 10% sodium dodecyl sulfate (SDS, final concentration, Pierce Thermo Fisher Scientific, Pittsburgh, PA) and vortexed vigorously to eliminate protoplasts, then OD_600_ was measured. Degradation was additionally verified through colony enumeration. The percent sensitivity of lactobacilli to lysozyme digestion was calculated using the following equation [27]:

$$\text{Sensitivity}\left(\%\right)=\left(\text{OD}\;600{\;}_{0\;\text{h}}-\text{OD}600_{1\;\text{or}\;2\text{h}}\right)/\text{OD}600_{0\;\text{h}}\;\text{x}\;100$$

### Human NOD2 activation

2.8 × 10^8^ CFU/mL of live or UV-killed lactobacilli were suspended with or without 100 μg/mL lysozyme from chicken egg white (L7651, Sigma-Aldrich), incubated aerobically at 37 °C for 2 h, and samples frozen at—80 °C. Lactobacilli digest samples were thawed and transfected into HEK-Blue hNOD2 cells (InvivoGen, San Diego, CA) using a calcium phosphate protocol. Specifically, lactobacilli digest samples were centrifuged, separated into soluble and insoluble fractions, and the insoluble product suspended in 80 μL of sterile water (Gibco). Sterile filtered 2M calcium chloride (10.2 μL) was added to 80 μL of either soluble or suspended insoluble product, added drop-wise to an equal volume of sterile filtered 2X HBSS (pH 7.05) while vortexing, and incubated at room temperature for 30 minutes. HEK-Blue hNOD2 cells were seeded at 7.5 × 10^4^ cells per well and transfected with the lactobacilli digest products in triplicate (final volume of 200.1 μL) in a 96-well format. Positive control L18-MDP (200 ng/mL, InvivoGen) and negative controls of sterile water and Pam_2_CSK_4_ (100 ng/mL, InvivoGen) were treated similarly. Activation of hNOD2 was assessed through NFκB/AP-1 activation of the stably transfected secreted embryonic alkaline phosphatase (SEAP) reporter after 44–48 hours of co-culture. SEAP concentration in the cell supernatants was evaluated using the chemiluminescent SEAP Reporter Gene Assay (Roche Diagnostics Corporation, Indianapolis, IN) per the manufacturer’s protocol. Sample means were calculated and expressed as fold difference over the sterile water control.

### Bone marrow derived macrophage isolation

Femoral, tibial, and humeral bones were dissected bilaterally and marrow was flushed and pooled on a per mouse basis. Bone marrow progenitor cells were cultured in petri dishes with 10% L-929 conditioned medium (supernatant from confluent L-929 (ATCC CCL-1) of less than 6 passages) cultures tested to be free of *Mycoplasma* and TLR2, -1/2, -2/6, -4, -5, -9, or NOD2 activating capacity (HEK293 TLR cell lines, InvivoGen). The base macrophage medium was DMEM/F12 supplemented with 10% FBS, 10mM L-glutamine, 100 U/mL penicillin, and 100 μg/mL streptomycin (Invitrogen, Carlsbad, CA). Medium was exchanged at day three and bone marrow derived macrophages (BMDM) were harvested using enzyme-free HBSS Cell Dissociation Buffer (Life Technologies) and manual disruption. BMDM purity was confirmed to be > 95% by F4/80 antibody (BioLegend, San Diego, CA) labeling [28].

### Phagocytosis assay and flow cytometry

BMDM (1.75 × 10^5^ cells/200 μL) were cultured for 24 h with medium only, Pam_2_CSK_4_ TLR2/6 agonist (100 ng/mL, InvivoGen), or CellTrace Violet labeled lactobacilli (10 CFU per 1 cell) in a 96-well plate. Supernatants and cells were harvested at 2 and 24 h. Cells were washed in Hank’s Balanced salt solution (HBSS) through diminishing concentrations of EDTA to dislodge extracellular lactobacilli (20mM, 10mM/10% FBS, 5mM/5% FBS, final 1.5mM EDTA-HBSS) on ice. BMDM Fc receptor was blocked (FcBlock; Miltenyi Biotec, San Diego, CA) and cells labeled with macrophage specific PE-anti-mouse F4/80, APC-anti-mouse CD11b, and PE/Cy7 anti-mouse I-A/I-E (BioLegend) followed by 7-aminoactinomycin (7-AAD) staining and fixation with 1% paraformaldehyde containing 5 μg/mL actinomycin D (Sigma-Aldrich) to prevent residual 7-AAD staining. Analysis was conducted on an LSR II flow cytometer with FACSDiva (BD Biosciences, San Jose, CA) and FlowJo software (Ashland, OR).

For immunization studies, freshly isolated B lymphocyte populations were analyzed via flow cytometry after undergoing Fc receptor blocking (BD Biosciences), staining with anti-mouse CD45-FITC, CD38-PECy7, CD19-Pacific Blue, and subsequent 7-AAD (all BioLegend), as previously described [16]. All cells were analyzed with Beckman Coulter Gallios Flow Cytometer (FL1-10) and FlowJo software (Ashland, OR).

### Fluorescence microscopy

Duplicate BMDM co-cultures were performed as reported for the phagocytosis assay, except BMDM were seeded onto chamber slides prior to co-culturing with CFSE labeled lactobacilli [29]. At 2 or 24 h, medium was removed, cells washed with PBS, and free aldehydes quenched with 50mM NH_4_Cl/PBS. After another PBS wash, cells were permeabilized with 1% Triton X-100/PBS, blocked with 1% bovine serum albumin/PBS, and cellular actin labeled with 5 U/mL Alexa Fluor 555 phalloidin (Life Technologies). Cells were mounted with VECTASHIELD mounting medium containing DAPI (4’,6-diamidino-2-phenylindole, Vector Laboratories, Burlingame, CA) and evaluated using a Leica DM5000B fluorescence microscope.

### Murine immunization: Sample preparation and cell isolation

C57BL/6J mice and BALB/cJ mice were intragastrically immunized as previously described [6, 20]. Briefly, C57BL/6 mice were immunized with buffered solution only or 2 × 10^9^ CFU of recombinant *Lactobacillus* (NCK1895 or NCK2166) on three consecutive days at week 0, 2, and 4; at week 6, mice were sacrificed. Similarly, BALB/c mice were immunized with three consecutive daily doses of 5 × 10^9^ CFU NCK1895 or GAD31 at two week intervals for a total of 18 immunizations. To normalize the gastrointestinal microbiome between BALB/cJ and *Nod2*-/- BALB/c mice bred in different locations, the cecal contents of a donor mouse from each strain were transferred to the opposing strain as previously described [30]. Briefly, 7–8 week old donor cecal contents were removed and suspended in 50 volumes of sterile PBS (Gibco); recipient mice were gavaged with 250 μl. The procedure was repeated 24 h later. Additionally, the mouse cages from opposing strains were exchanged after 3 days and were not changed for a minimum of 3 days to permit cross-environmental exposure.

For evaluation of antibody responses, serum, vaginal wash, and fecal pellets were collected prior to the first immunization and each boost [20]. At sacrifice, spleen, intestine, female reproductive tract (FRT), mesenteric lymph node (MLN), Peyer’s patches (PP), and cecal contents were also harvested [6]. The vaginal vault was lavaged with 100 μl of PBS and the insoluble debris removed via centrifugation. Fecal pellets were suspended at 100 mg/mL in PBS with 10% goat serum, 2X proteaseArrest (G-Biosciences, St. Louis, MO) and 0.1% Kathon, homogenized (FastPrep-24, MP Bio 4.0 m/s for 20 s for three cycles), and supernatant collected post-centrifugation. Cecal contents were processed by the same method as fecal pellets, but without proteaseArrest. Blood was periodically collected from tail vein or terminally via cardiac puncture. All samples were aliquoted and stored at– 80 °C until analysis. Single cell suspensions of lymphocytes were isolated from the MLN, PP, spleen, colon, and FRT for ELISpot and B cell phenotyping. MLN were pushed through a 40 μm cell strainer with a syringe plunger. PP and spleen were dissociated in 5 mL cytotoxic T lymphocyte (CTL) medium using the gentleMACS Octo Dissociator (Miltenyi setting m_spleen_01_01), followed by incubation in ACK (ammonium chloride potassium) buffer to lyse red blood cells in the spleen samples [26]. Colon and FRT were manually cleaned of mucus and debris in PBS, 1mM dithiothreitol, 5 mM EDTA, cut into ~1cm pieces, and digested with Liberase TM (125 μg/mL; Roche Molecular) and DNAse I (grade II 100 μg/mL; Roche Molecular) in digestion base media (RPMI containing 5 μg/mL gentamicin, 100 U/mL penicillin, and 100 μg/mL streptomycin (Invitrogen, Carlsbad, CA) for 30 or 60 minutes, respectively, with constant tissue rotation at 37 °C. Tissue was mechanically disrupted prior to and after enzymatic digestion using the gentleMACS dissociator (Program B and m_liver_02). Post-digestion, tissue was passed through a 70 μm filter; lymphocytes were isolated using a Percoll gradient and suspended in CTL media as described previously [16]. After dissociation, all cells were washed with CTL media then passed through a 40 μm strainer. Small intestine *ex vivo* culture was performed as previously described to assess tissue cytokine production [31, 32]. Briefly, small intestine was opened longitudinally, irrigated with PBS, and shaken for 30 minutes at 240 rpm, room temperature (RT), in digestion base media. Tissue was subsequently blotted, weighed, diced, and distributed into a 24-well plate (0.05 g/well) with 150 μl CTL media. After 24 h incubation at 37 °C, 5% CO_2_, supernatants were harvested and stored at—80 °C until analyzed in cytokine assays.

### Cytokine analysis

Supernatants from 24 h BMDM-lactobacilli co-culture were evaluated for IL-6, tumor necrosis factor (TNF)-α, IFN-γ, IL-10, IL-12 (p70), IL-17, IL-4, granulocyte-macrophage colony-stimulating factor (GM-CSF), IL-1β, IL-12 (p40), chemokine (C-X-C motif) ligand 1 (CXCL1 or KC), and macrophage inflammatory protein (MIP)-1α using a customized 12-plex Milliplex MAP mouse cytokine/chemokine magnetic bead assay (Millipore, Billerica, MA) per manufacturer’s protocol. The lower and upper limits of detection for the assay (pg/mL) were 3.19–10012.78 (IL-6), 3.1–1896.06 (TNF-α), 3.14–10094.09 (IFN-γ), 3.11–9999.54 (IL-10), 2.77–10020.87 (IL-12 (p70)), 3.23–8964.25 (IL-17), 3.18–7982.37 (IL-4), 9.65–9546.41 (GM-CSF), 2.14–7194.07 (IL-1β), 2.7–9999.03 (IL-12 (p40)), 3.08–10628.11 (CXCL1), and 3.28–9922.54 (MIP-1α). The 24 h small intestine *ex vivo* culture supernatants were evaluated using a customized 14-plex Milliplex MAP mouse TH17 cytokine magnetic bead assay (Millipore) per manufacturer’s protocol. Only IL-1β data are reported; lower and upper limits of detection for the assay were 15.08–13893.06 pg/mL.

### Colorimetric ELISA for murine antibody detection

For detection of HIV-Gag, HIV-MPER, or lactobacilli SlpA-specific antibody from serum, vaginal wash, and cecal contents, plates (Maxisorp; Nunc, Rochester, NY) were coated overnight at RT with AT-2 inactivated HIV IIIB lysate (1.9 ng/mL) in carbonate buffer (pH 9.6), synthetic 17-mer MPER peptide GNEQELLELDKWASLWN (Bio-Synthesis Inc, Lewisville, TX; 1 μg/mL), or SlpA isolated from NCK1909 (1 μg/mL) [20]. Plates were washed five times with PBS containing 0.05% Tween-20 (PBST) for HIV ELISAs, or once for SlpA, then blocked with 1% bovine serum albumin (BSA)/PBS for 1 h at RT, and washed as before. Controls and samples diluted in 1% BSA/PBST were added and incubated for 2 h at RT, washed five times in PBST, and incubated with horseradish peroxidase (HRP)-conjugated anti-mouse IgG (Pierce Thermo Fisher Scientific for HIV-Gag and Cell Signaling Technology for MPER and SlpA) or IgA (Bethyl Laboratories, for vaginal wash and cecal contents) antibody added at 40 ng/mL or 20 ng/mL, respectively, for 1 h at RT. Plates were washed 5 or 7 times and developed with TMB peroxidase substrate solution (SeraCare, Milford, MA). The reaction was stopped with an equal volume of 1N sulfuric acid and absorbance measured on a plate reader (BioTek, Winooski, VT) and reported as 450 nm-570 nm or 450 nm. A background curve utilizing the average absorbance data obtained from C57BL/6 control animals (buffer or NCK1895 immunized mice) was generated for each mouse strain genotype (*Nod2*+/+ or *Nod2-/-*) in the y = cx^b^ or y = mx+b format for HIV-specific and SlpA-specific ELISA, respectively. Criterion for antigen-specific seroconversion was determined as absorbance 2-fold greater than the respective control standard curve value at the same dilution. For BALB/c endpoint titer, mean value plus three standard deviations of buffer control mice was used as the cut-off.

### Total IgA ELISpot assay

IgA secreting cells were detected via ELISpot as previous described [6]. MultiScreen assay plates (Millipore) were aseptically treated with 50 μL of 70% ethanol and washed five times with 200 μL distilled water (Gibco). Plates were coated with 100 μL of 10 μg/mL α-mouse IgA (MabTech, Mariemont, OH) and incubated overnight at 4 °C. Plates were washed as prior, wells blocked with 200 μL of CTL media for a minimum of 2 h at 37 °C, and 1 × 10^4^ cells per well added. Lymphocytes were incubated for 20 h at 37 °C and plates washed 6 times with PBST prior to the addition of filtered α-IgA-biotin in 0.5% FBS/PBS detection antibody (MabTech) for 2 h at RT. For detection, plates were washed 3 times with PBST, 3 times with PBS, and filtered 3,3’,5,5’-Tetramethylbenzidine applied for 2 min at RT. Plates were washed 10 times with water and air dried. Spots were counted in an ImmunoSpot analyzer (Cellular Technology Limited).

### IFN-γ ELISpot assay

IFN-γ secreting cells were detected via ELISpot as previously described [6, 20]. MultiScreen assay plates (Millipore) were treated with 15 μL of 35% ethanol and washed three times with sterile PBS (Gibco). Plates were coated with 50 μL of 10 μg/mL α-mouse IFN-γ (AN18, Mabtech) and incubated overnight at 4 °C. Plates were washed as before, wells blocked with 200 μL of CTL medium for a minimum of 2 h at 37 °C, 2.5 × 10^5^ lymphocytes were added into wells with or without 10 ng/mL (final concentration) of AT-2 inactivated HIV-1 IIIB viral lysate or 10 μg/mL synthetic 17-mer MPER peptide, GNEQELLELDKWASLWN in CTL medium; additional wells included 50 ng/mL phorbol-myristate-acetate and 300 ng/mL ionomycin as a positive control [33]. Lymphocytes were incubated over two nights at 37 °C in 5% CO_2_, then washed 6 times with PBST, and 100 μL filtered biotinylated IFN-γ detection antibody (Mabtech) was added at 1 μg/mL in 0.5% FBS/PBS for 3.5 h at RT. Plates were washed 6 times with PBST, and streptavidin-HRP (1 μg/ml, Pierce Thermo Fisher Scientific) in 5% FBS/PBS was added for 1 h at RT, followed by 3 PBST and 3 PBS washes. Plates were developed with 3-amino-9-ethylcarbazole substrate (Sigma-Aldrich) for 10 min at RT. Spots were counted in an ImmunoSpot analyzer (Cellular Technology Limited).

### Statistics

*In vitro* co-culture data were analyzed using two- and three-factor repeated measures models: mouse-type, bacterial treatment, and bacterial uptake (bacteria^+^ or bacteria^-^/exposed). Means were compared within and across time points and response variables were modeled with the PROC MIXED statement in SAS software version 4.3 (Cary, NC) using restricted maximum likelihood (ReML) parameter estimation with Sattherthwaite approximation for degrees of freedom. Means between treatments were assessed using the “slice” statement with a Tukey-Kramer adjustment, whereas means compared to controls used the Dunnett’s adjustment. However, as the differences between NCFM and NCK2025 and NCK2031 were considered *a priori*, no adjustment was made for multiple comparisons. In order to satisfy the assumptions for analysis of variance (ANOVA), flow cytometric data expressed as a percentage were transformed using arcsine of the square root; a log_10_ transformation was used for flow cytometric MFI data along with cytokine data. In order to perform statistics on cytokine concentrations that were below the assay lower limit of detection (LLOD), values were assigned that were half of the LLOD [34]. If more than half of the cytokine responses were below the LLOD, the treatment or mouse-type was excluded from the analysis; thus, 5 cytokines were excluded from the analysis (GM-CSF, IL-12 (p70), IFN-γ, IL-17, and IL-4).

*In vivo* data were analyzed using GraphPad Prism 6.0 and 7.0. The analysis of variance general linear model was used to compare treatment data for flow cytometric data, with a Bonferroni’s Multiple Comparison adjustment. A Kruskal-Wallis test of analysis of variance was applied to endpoint titer ELISA, ELISpot, and cytokine data, with a Dunn’s multiple comparison test post-hoc, as data were not normal. In order to perform statistics on cytokine concentrations that were below the assay LLOD, values were assigned that were half of the LLOD [34]. A two-tailed Pearson r correlation test was performed on ELISpot and flow cytometric data; a two-tailed Spearman r correlation test was performed on antibody and histology scores. For time-course ELISA data, a multiple unpaired t-test, corrected for multiple comparisons with Holm-Sidak method, was performed. ELISA seroconversion data and histological grouping data were compared using a Chi-square test with a two-tailed Fisher’s exact test. Statistical significance was set at *P* < 0.05 for all data, using adjusted p-values as noted.

## Results

### NOD2 activation by lactobacilli influences phagocytosis and phenotypic activation of macrophages

**Fig 1:**
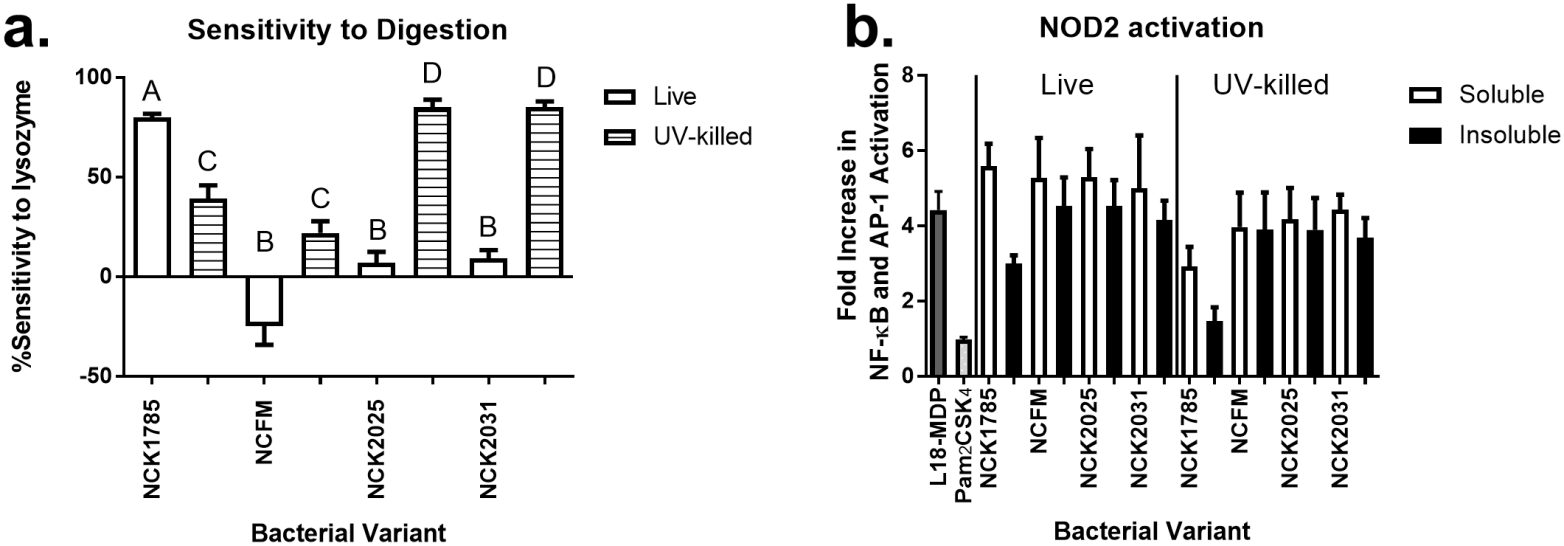
Variable sensitivity of lactobacilli to lysozyme digestion and NOD2 activation. Live or UV-killed lactobacilli were digested with lysozyme for 2 h and OD_600_ assessed at t = 0 and 2h. Duplicate 2 h lysozyme digests were separated into soluble or insoluble portions via centrifugation and transfected into HEK hNOD2 cells. Positive control, L18-MDP, and negative control, Pam_2_CSK_4_, were treated similarly. (a) Data (mean with SEM) is displayed as % sensitivity to lysozyme digestion for each bacterial variant. Means with the same letter are not significantly different (*P* > 0.05) from each other. (b) Data (mean with SEM) is reported as the fold increase of NFκB/AP-1 activation over negative control as determined by chemiluminescent detection of culture supernatant SEAP after 44–48 h co-culture. N = 3–8 samples per group.

NOD2 and TLR2 have been implicated as key modulators of the innate immune response to lactobacilli, and TLR2 activation by *L*. *gasseri* (strain NCK1785) and *L*. *acidophilus* (strain NCFM) has been demonstrated (Table 1) [15, 16, 35]. Ordinarily, lactobacilli taken up by macrophages are exposed to hydrolytic enzymes in the phagolysosome that digest PGN into constitutive components, including MDP. To demonstrate the ability of MDP from different lactobacilli to activate NOD2, we exposed HEK293 cells stably transfected with human NOD2 linked to a secreted embryonic alkaline phosphatase (SEAP) reporter gene to lysozyme-digested lactobacilli. Despite strain-dependent variability in the susceptibility of bacteria to lysozyme digestion, the soluble and insoluble fractions of each lactobacilli species, strain, and mutant strongly activated NOD2 (Fig 1) to a degree not significantly different from the positive control (NOD2 ligand, L18-MDP).

**Fig 2:**
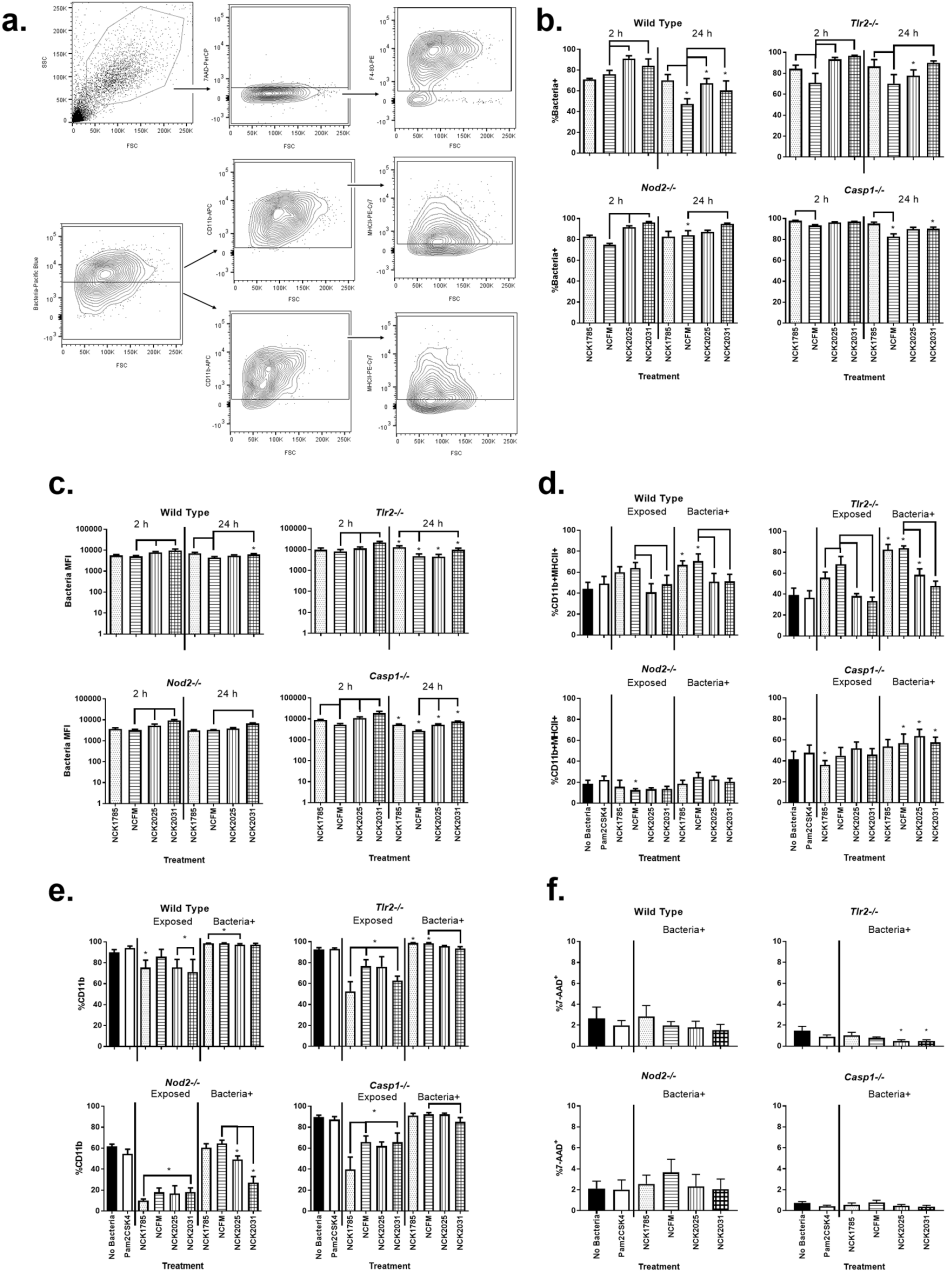
BMDM phenotype after 24 h co-culture with lactobacilli. BMDM derived from wild type C57BL/6 or *Tlr2-/-*, *Nod2-/-*, or *Casp1-/-* mice, were co-cultured with either negative control (no bacteria), TLR2/6 agonist (Pam_2_CSK_4_), or Cell Trace Violet stained lactobacilli bacteria (10 CFU bacteria per 1 cell) as indicated for 2 or 24 h prior to harvest for flow cytometric analysis. (a) Cells were gated on 7-AAD^-^F4/80^+^cells to assess purity and then 7-AAD^-^ BMDM subdivided into Bacteria^+^ (Bacteria +) or Bacteria^-^ (Exposed) populations, and the percentage of CD11b^+^, and subsequent MHCII^+^ cells, determined. Data (mean with SEM) represented as the (b) percentage of Bacteria^+^ BMDM cells or (c) MFI (median fluorescence intensity) of respective bacteria within Bacteria^+^ BMDM cells across treatments and BMDM genotypes. Asterisk (*) indicates statistically significant difference (*P* < 0.05) between 24 h data and corresponding 2 h treatment; bracket denotes significance between respective treatments within the same group and timepoint. 24 hr BMDM phenotype data displayed as (d) the percentage (mean with SEM) of CD11b^+^MHCII^+^, (e) CD11b^+^, and (f) 7-AAD^+^ BMDM cells across bacterial treatments and BMDM genotypes. Asterisk (*) denotes a significant difference (*P* < 0.05) between respective treatments and no bacteria control. Bracket alone denotes significant difference (*P* < 0.05) between treatments within a BMDM genotype. N = 5–6 separate experiments.

Phagocytosis and phagosome maturation are required for immune recognition of microbiota and have been correlated with macrophage cytokine production [27, 36]. To determine the necessity of relevant PRR and downstream pathways for macrophage phagocytosis, Cell Trace Violet-stained lactobacilli were co-cultured with wild type (WT), *Tlr2-/-*, *Nod2-/-*, or *Casp1-/-* BMDM for 2 or 24 h and bacterial uptake was evaluated using flow cytometry (Fig 2a and 2b). The percentage of WT BMDM containing NCK1785 or NCFM was similar at 2 h; however, significantly fewer WT BMDM contained NCFM after 24 h (*P <* 0.0001, Fig 2b). NCFM mutants lacking LTA (NCK2025) or SlpB/X (NCK2031) were found in a significantly higher percentage of WT BMDM than was NCFM at 2 h (*P* < 0.0001 and *P* = 0.012, respectively) and 24 h (*P* = 0.003 and *P* = 0.030, respectively). Compared to WT BMDM, *Tlr2-/-* and *Nod2-/-* BMDM did not eliminate NCFM as efficiently over 24 h, and a greater percentage of *Casp1-/-* BMDM contained NCFM at 2 and 24 h (*P* = 0.017). *Tlr2-/-* and *Casp1-/-* BMDM also had altered NCK1785 and NCFM load, as evidenced by significantly decreased bacterial MFI from 2 to 24 h (*P* = 0.036 and *P* = 0.0002 in *Tlr2-/-* and *P* = 0.002 and *P* = < 0.0001 in *Casp1-/-*, respectively; Fig 2c).

**Fig 3:**
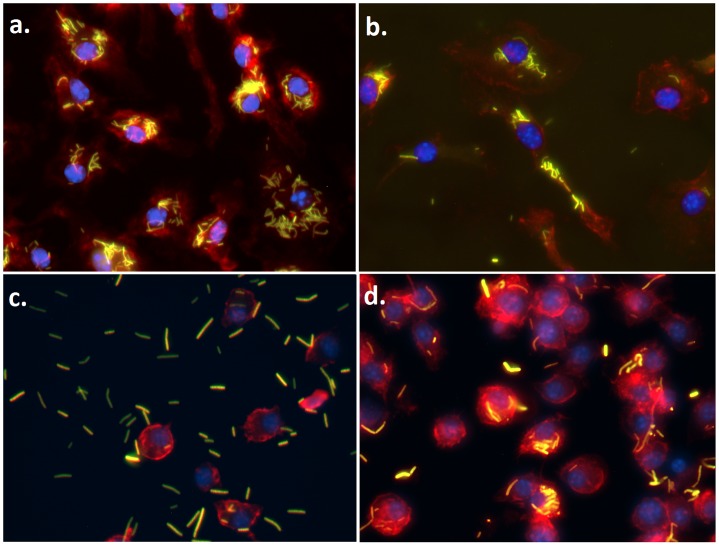
BMDM morphology in culture. BMDM from (a) wild type C57BL/6, (b) *Tlr2-/-*, (c) *Nod2-/-*, and (d) *Casp1-/-* mice were co-cultured with CFSE-labeled lactobacilli (10 CFU per 1 cell; green). After 24 h, media was removed, cells were washed and stained with actin-specific phalloidin (red) and DNA-specific DAPI (blue), and subsequently evaluated using fluorescence microscopy. Representative images are shown. N = 6 separate experiments.

The innate immune signaling involved in BMDM activation was assessed by flow cytometry after 24 h co-culture with lactobacilli by staining for cell surface CD11b and major histocompatibility complex class II (MHCII) (Fig 2). In WT BMDM, CD11b and MHCII expression were significantly increased after co-culture with NCK1785 or NCFM (*P* = 0.007 or *P* = 0.040, respectively). Upregulation of these markers after co-culture with NCK2025 and NCK2031 was significantly lower than with NCFM (*P* = 0.0003 and *P* = 0.0004, respectively; Fig 2d). The pattern of CD11b and MHCII expression and upregulation was similar in *Tlr2-/-* BMDM (*P* < 0.0001 for all). Baseline expression of CD11b and MHCII was lower in *Nod2-/-* BMDM as compared to WT BMDM (*P* < 0.001 and *P* = 0.0006), and treatment with lactobacilli did not significantly increase the expression of these markers (Fig 2d and 2e). Consistent with the lack of phenotypic activation, *Nod2-/-* BMDM lacked morphologic features of activation including cell adhesion and pseudopodia that were observed in WT and *Tlr2-/-* BMDM (Fig 3). The *Casp1-/-* BMDM also lacked morphologic features of activation but showed insignificant increases in CD11b and MHCII after uptake of lactobacilli (Fig 2d). Notably, the percentage of 7-AAD^+^ cells, indicative of pyroptosis, was not significantly elevated between BMDM across treatment based upon genotype, including *Casp1-/-* (Fig 2f) [37].

### Inflammatory cytokine induction by *Lactobacillus spp*. is dependent on NOD2 and caspase-1 activation

**Fig 4:**
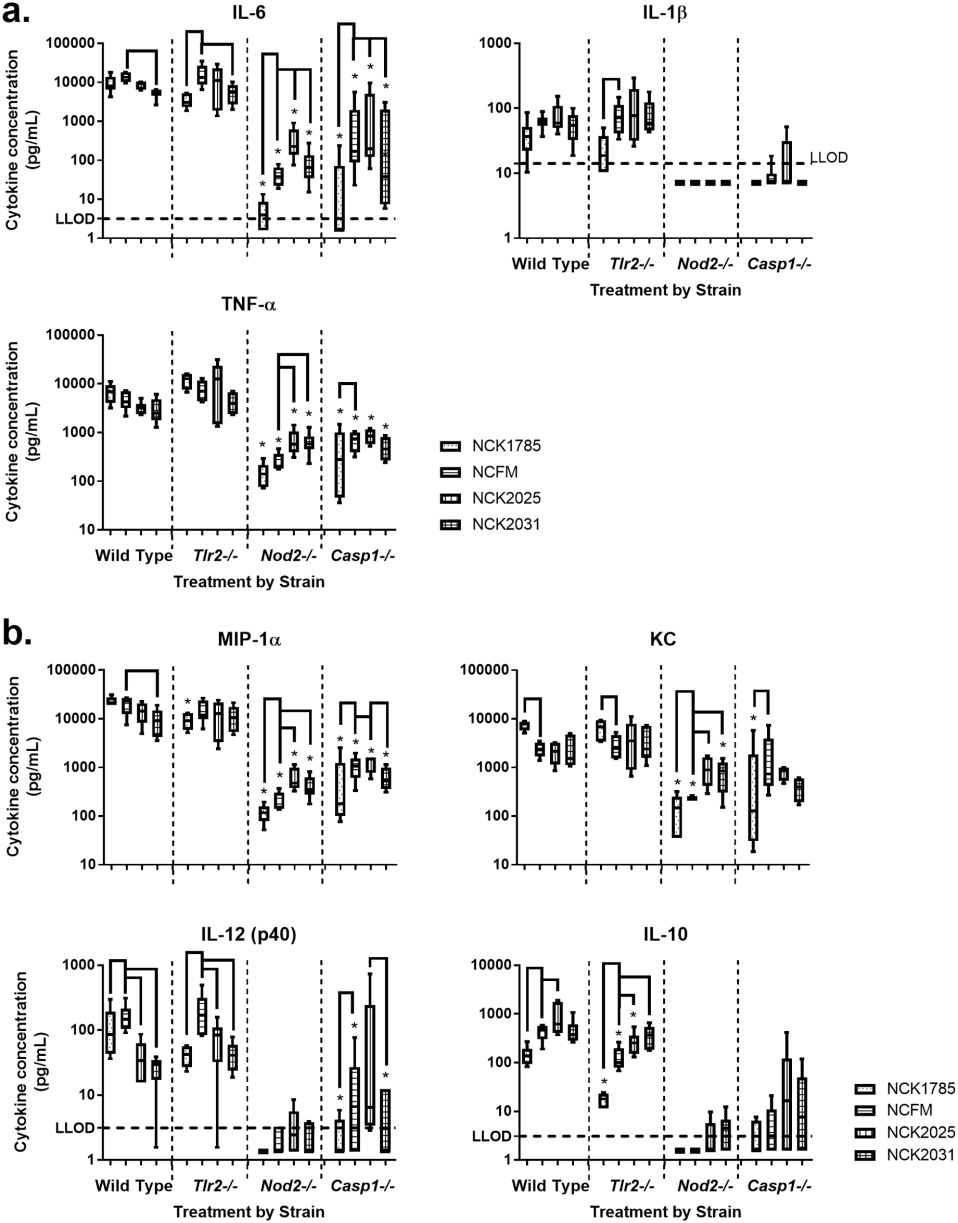
Cytokine and chemokine production by BMDM after 24 h co-culture with lactobacilli. BMDM from wild type C57BL/6, *Tlr2-/-*, *Nod2-/-*, or *Casp1-/-* mice were co-cultured with NCK1785, NCFM, or NCFM mutants (NCK2025 or NCK2031) (10 CFU bacteria per 1 cell) for 24 h and culture supernatants harvested for cytokine and chemokine quantification using a 12-plex Milliplex MAP mouse assay. Box (percentiles) and whisker (minimum and maximum) data for (a) IL-6, IL-1β, and TNF-α and (b) MIP-1α, KC, IL-12 (p40), and IL-10 displayed for lactobacilli across BMDM genotype. Asterisk (*) denotes a significant difference (*P* < 0.05) in cytokine concentration between the respective knock-out BMDM and wild type BMDM treatment. Bracket denotes significant difference (*P* < 0.05) between treatments within a BMDM genotype. N = 5–6 separate experiments.

To establish the cytokine profile of WT BMDM in response to lactobacilli, 24 h co-culture supernatants were harvested for quantitative cytokine and chemokine evaluation using multiplex technology. No treatment induced significant production of GM-CSF, IL-12 (p70), IFN-γ, IL-17, or IL-4. Co-culture of NCK1785 or NCFM increased secretion of IL-6, IL-1β, TNF-α, and MIP-1α in WT BMDM; there were no significant differences between treatments (Fig 4). Co-culture of WT BMDM with NCK1785 yielded significantly higher concentrations of keratinocyte chemoattractant (KC, *P* = 0.0006), but significantly lower concentrations of IL-12 (p40) and IL-10 (*P* = 0.028 and *P* < 0.0001, respectively), compared to NCFM. On the other hand, co-culture of WT BMDM with NCK2031 resulted in significantly lower concentrations of IL-6, MIP-1α, and IL-12 (p40) compared to NCFM (*P* = 0.015, *P* = 0.001, *P <* 0.0001, respectively), and with NCK2025 resulted in significantly lower concentration of IL-12 (p40) but significantly higher concentration of IL-10 compared to NCFM (*P* = 0.002 and *P* = 0.013, respectively). The lack of SlpB/X or LTA expression by NCFM did not alter IL-1β, TNF-α, or KC production by WT BMDM.

To evaluate the requirement for TLR2, NOD2, and caspase-1 for cytokine induction by lactobacilli strains, *Tlr2-/-*, *Nod2-/-* and *Casp1-/-* BMDM were treated similarly to the WT BMDM described above. In the absence of *Nod2* or *Casp1*, all cytokines and chemokines measured (IL-6, IL-1β, TNF-α, MIP-1α, IL-10, IL-12 (p40), and KC) after co-culture with NCK1785 were significantly lower than observed with WT BMDM (Fig 4; *P* < 0.0001). Similarly, *Nod2-/-* and *Casp1-/-* BMDM produced significantly lower concentrations of almost all cytokines/chemokines evaluated in response to NCFM (*P* < 0.0001 where indicated, except TNF-α for *Casp1-/-*, where *P* = 0.0003). Additionally, *Tlr2-/-* BMDM expressed significantly lower concentrations of MIP-1α and IL-10 after co-culture with NCK1785 (*P* = 0.018 and *P* < 0.0001, respectively), and lower concentrations of IL-10 after co-culture with NCFM (*P* = 0.0001). Interestingly, while NCK2025 induced greater IL-10 expression by WT BMDM (*P* = 0.013), IL-10 expression was diminished in the absence of TLR2 signaling (*P* = 0.0004).

### NOD2 signaling is required for antigen-specific systemic humoral responses after intragastric immunization of C57BL/6 mice

**Fig 5:**
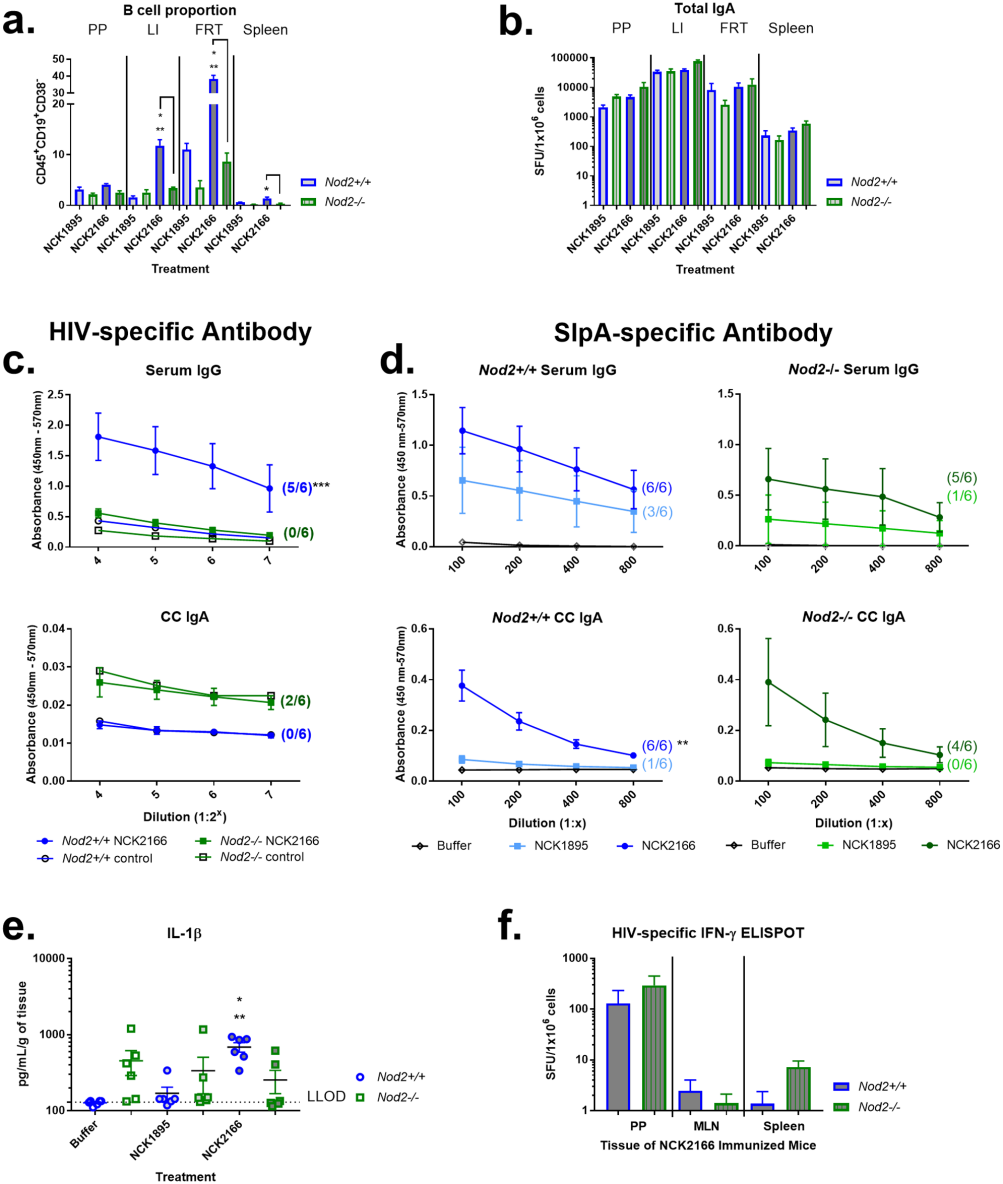
Differential IgG response of immunized C57BL/6 mice based upon *Nod2* expression and IL-1β. Wild type *Nod2+/+* or *Nod2-/-* C57BL/6 mice were immunized as described in methods with buffer only, NCK1895, or NCK2166. At sacrifice, lymphocytes were isolated from Peyer’s patches (PP), large intestine (LI), female reproductive tract (FRT), and spleen; and analyzed by flow cytometry. (a) Data represented as mean percentage (and SEM) of 7-AAD^-^CD45^+^CD19^+^CD38^-^ B cells across tissue type from NCK1895 and NCK2166 immunized mice. (b) Lymphocytes were additionally applied to total IgA ELISpot assay for 20 h of incubation without stimulation. Data represented as spot forming units (SFU) per 1 × 10^6^ live (7AAD^-^) B cells (CD45^+^CD19^+^ and/or CD45^+^CD38^+^). At sacrifice, serum and cecal contents were harvested, serially diluted, and applied to antigen-specific colorimetric ELISA, using (c) HIV IIIB lysate (d) or lactobacilli SlpA as antigen, with α-IgG or -IgA detection antibody. Data are represented as absorbance (mean with SEM) across serial dilutions along with the standard curve value (open circle or square) derived from control. Seroconversion of immunized animals was defined as 2-fold absorbance over the standard curve at 2 or more dilutions. The number of NCK2166 or NCK1895 immunized animals that met this criterion is shown in parentheses. (e) Small intestine *ex vivo* cellular supernatants were utilized in a customized 14-plex Milliplex MAP mouse TH17 cytokine magnetic bead assay and results reported as pg/mL of cytokine per g of tissue cultured. Only IL-1β cytokine data (mean with SEM) is shown with assay lower limit of detection (LLOD) indicated by horizontal dashed line. (f) Lymphocytes isolated at sacrifice from PP, MLN, and spleen were stained for flow cytometry or incubated in duplicate with or without AT-2 inactivated HIV IIIB lysate for IFN-γ ELISpot assay. Spots were counted after 2 nights of incubation and data (mean with SEM) represented as the difference of spot forming units (SFU) from HIV IIIB stimulated and unstimulated cells per 1 × 10^6^ live, CD45^+^ cells. Asterisk (*) indicates significance (*P* < 0.05) of difference between treatment group and buffer-only group of the same *Nod2* genotype status; (**), between treatment group and NCK1895-treated group of the same *Nod2* genotype status; (***) between treatment group and *Nod2-/-* mice receiving same treatment. N = 4–6 animals per group.

NOD2 signaling plays a role in regulating the gastrointestinal microbiome and shaping the mucosal immune response, including humoral immunity [38–40]. Since *Nod2* was required for NCFM immune activation of macrophages, we sought to determine whether our NCFM-derived oral immunization strategy expressing an HIV-1 model antigen (Gag) was dependent on NOD2 signaling *in vivo* [6, 20]. WT *Nod2+/+* and *Nod2-/-* C57BL/6 mice were immunized with buffer alone, NCK1895, or NCK2166 by oral gavage (Table 1). Lymphocytes were collected at sacrifice from the FRT, large intestine (LI), PP, MLN, and spleen for flow cytometric phenotyping and determination of IgA secretion via ELISpot. While the percentage of CD45^+^CD19^+^CD38^-^ B cells was significantly larger for NCK2166-immunized WT mice than for NCK2166-immunized *Nod2-/-* mice for most mucosal and systemic tissues (*P* < 0.05), this did not correspond with differences in IgA-producing cells as measured via ELISpot (Fig 5a and 5b). To determine whether NOD2 status dictated differential antigen-specific IgA levels, cecal contents and vaginal lavage samples were collected from mice at sacrifice and mucosal anti-Gag IgA was measured by ELISA. Gag-specific IgA levels in cecal contents were not statistically significantly different between NCK2166-immunized *Nod+/+* and *Nod-/-* mice (Fig 5c). After immunization with NCK2166, only 2 of 6 *Nod2-/-* mice and no *Nod2+/+* mice were positive for antigen-specific IgA in cecal contents (Fig 5c). No mice were positive for antigen-specific IgA in vaginal washes. Conversely, Gag-specific IgG levels in sera were statistically significant, with antigen-specific serum IgG detected in 5 of 6 WT mice compared to 0 of 6 *Nod2-/-* mice. Antibody responses to the highly expressed surface layer protein A (SlpA) of *L*. *acidophilus* can serve as an endogenous bacterial antigen for comparison against recombinant antigen responses. We found no correlation between anti-SlpA IgA or IgG seroconversion and HIV-specific antibody responses in C57BL/6 mice (Fig 5d).

To determine whether the cytokine milieu present within the intestinal mucosa of immunized mice correlated with humoral responses, supernatants from cultured small intestine were evaluated in a customized 14-plex cytokine assay. In WT mice, IL-1β was significantly elevated in mice immunized with NCK2166 compared to animals immunized with either buffer only or NCK1895 controls (Fig 5e; *P* = 0.0096). No other differences of measured cytokines were observed. HIV-specific IFN-γ production was also evaluated via IFN-γ ELISpot, with or without stimulation with AT-2 inactivated HIV IIIB lysate. While there were detectable levels of HIV-specific IFN-γ producing cells across NCK2166 immunized mice, there were no significant differences between *Nod2+/+* and *Nod2-/-* mice (Fig 5f).

*Nod2-/-* mice have been shown to have an increased number of lymphoid follicles and PP with enhanced paracellular permeability, bacterial translocation, and altered bacterial killing and persistence [41, 42]. To evaluate inflammation and vector persistence amongst groups, tissue samples were harvested at sacrifice for routine histopathology (S1 Fig. Histological evaluation of large and small intestine in C57BL/6 mice) and MLN and ileal contents were collected and selectively cultured to quantify lactobacilli vaccine strains (S1 Table. Persistence of NCK2166 in immunized mice at endpoint). No histopathologic abnormalities were observed in the spleen from any animal. The numbers of abnormal tissue samples were not significantly different amongst groups, and there was no positive correlation detected between colonic inflammatory scoring and humoral responses (S1 Supporting materials and methods). Additionally, the infrequent persistence of NCK2166 was not *Nod2*-dependent and did not correlate with humoral responses.

### NOD2 signaling is required for antigen-specific systemic and mucosal humoral responses after intragastric immunization of BALB/c mice

**Fig 6:**
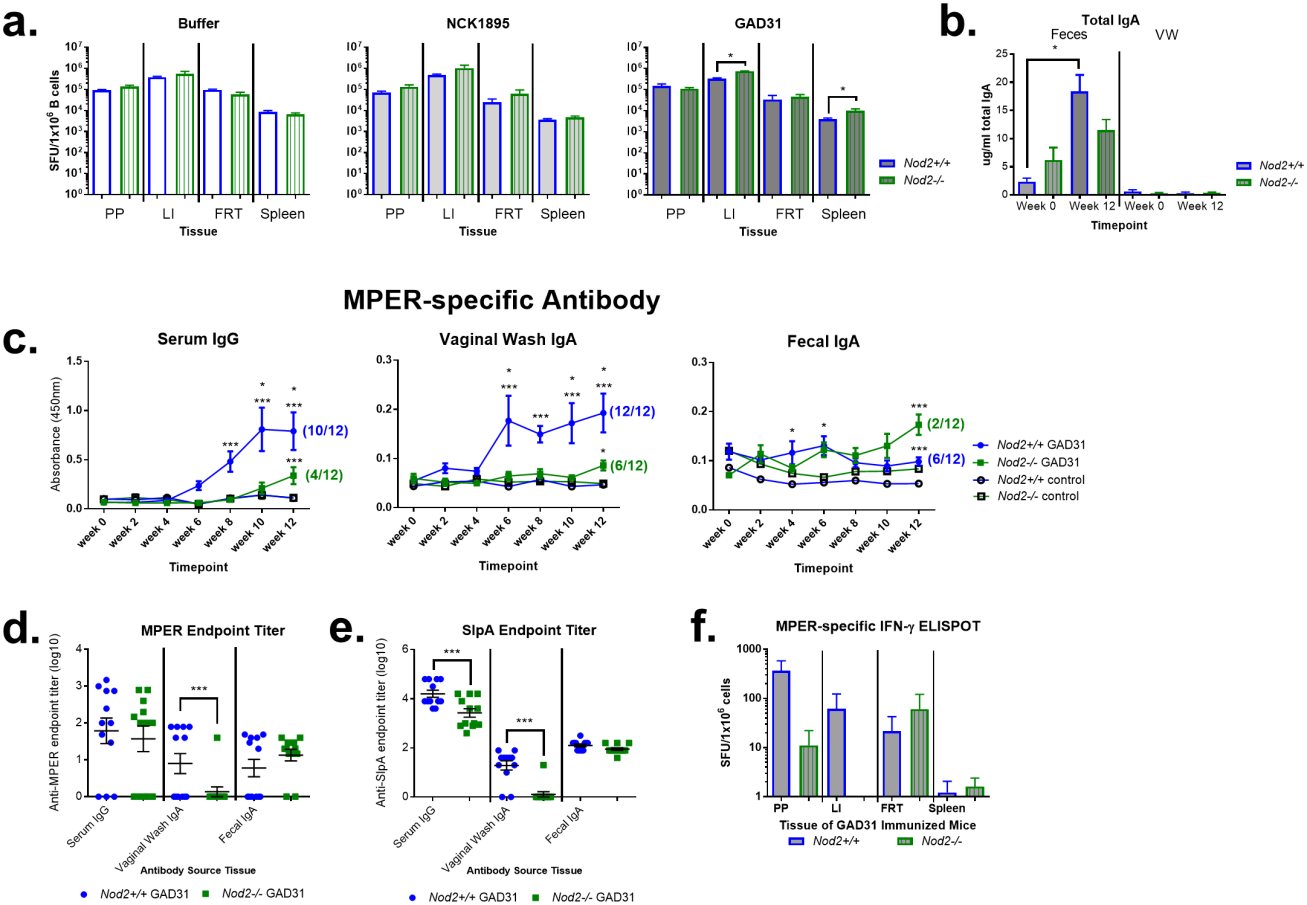
Differential IgG and IgA response of BALB/c immunized mice based upon *Nod2* expression. Wild type *Nod2+/+* or *Nod2-/-* BALB/c mice were immunized with buffer only, NCK1895, or GAD31. (a) At sacrifice, lymphocytes were isolated from Peyer’s patches (PP), large intestine (LI), female reproductive tract (FRT), and spleen; applied to total IgA ELISpot assay for 20 h of incubation without stimulation. Data represented as spot forming units (SFU) per 1 × 10^6^ live B cells (CD45^+^CD19^+^ and/or CD45^+^CD38^+^) across tissue per immunization group. N = 4–6 animals per group. (b) Fecal extract and vaginal wash were harvested from GAD31 immunized mice at week 0 and week 12/sacrifice and applied to a quantitative colorimetric ELISA to evaluate total IgA. Data (mean plus SEM) are represented as μg/mL of total IgA. Asterisk (*) indicates significance (*P* < 0.05) of difference between indicated treatment groups. N = 12 animals per group. At 0, 2, 4, 6, 8, 10, and 12 weeks/sacrifice serum, feces, and vaginal wash were harvested, serially diluted, and applied to antigen-specific colorimetric ELISA, using HIV MPER (c and d) or lactobacilli SlpA as antigen (e), with α-IgG or -IgA detection antibody. Data are represented as absorbance (mean with SEM) across time at 1:100 dilution for MPER-specific and 1:1000 for SlpA-specific serum and 1:10 for vaginal wash and feces. Average control values at the same dilution are represented as open circle or square. Seroconversion of immunized animals was defined as absorbance 3-fold greater than the average absorbance from control animals, and the number of GAD31 immunized animals that met this criterion is shown in parentheses; N = 6–12 animals per group (c). The same criterion was used for endpoint titer data, with values under detection limit assigned 0; N = 12 animals per group (d and e). (f) Lymphocytes isolated at sacrifice from PP, LI, FRT, and spleen were stained for flow cytometry or incubated in duplicate with or without HIV MPER for IFN-γ ELISpot assay. Spots were counted after 2 nights of incubation and data (mean with SEM) represented as the difference of SFU from HIV MPER stimulated and unstimulated cells per 1 × 10^6^ live, CD45^+^ cells; N = 4–6 animals per group. Asterisk (*) indicates significance (*P* < 0.05) of difference between treatment group and buffer-only group of the same *Nod2* genotype status; (***), between treatment group and *Nod2-/-* mice receiving same treatment.

As C57BL/6 mice have a decreased propensity for IgA class switching and a well-known Th1 versus Th2 cytokine bias in comparison to BALB/c mice, an alternative BALB/c model was pursued to determine if NOD2 signaling is required for both antigen-specific IgG and IgA responses [43]. *Nod2+/+* and *Nod2-/-* BALB/c mice were immunized with buffer only, NCK1895, or GAD31 (Table 1). GAD31 expresses HIV-1 membrane proximal external region (MPER) within the surface layer protein of NCFM and has previously been shown to induce MPER-specific mucosal IgA and systemic IgG responses in BALB/c mice [20]. Unlike C57BL/6 mice immunized with NCK2166, GAD31-immunized BALB/c *Nod2+/+* mice had significantly increased total IgA spot forming units (SFU) in the LI and spleen, as measured by ELISpot, in comparison to *Nod2*-/- mice (Fig 6a; *P* = 0.029 and *P* = 0.015, respectively). In addition, a significant increase in total fecal IgA, as measured by ELISA, was identified from 0 to 12 weeks in GAD31-immunized *Nod2*+/+ mice only (Fig 6b; *P* < 0.0001).

GAD31-immunized *Nod2+/+* mice had significantly more MPER-specific serum IgG, from week 8 to week 12 (*P* = 0.002 for week 8 and *P* < 0.0001 for week 10 and 12, respectively), and vaginal IgA, from week 6 to week 12, compared to *Nod2*-/- mice (*P* = 0.003, *P* = 0.010, *P* = 0.005, and *P* = 0.007, respectively; Fig 6c). The endpoint titer of MPER-specific vaginal IgA, but not serum IgG, at the terminal time point was significantly higher in *Nod2*+/+ than *Nod2*-/- mice (*P* = 0.002; Fig 6d). However, while the relative amount of MPER-specific fecal IgA in *Nod2*-/- was significantly greater than WT mice at week 12, only 2 of 12 *Nod2-/-* mice versus 6 of 12 *Nod2+/+* mice were positive for MPER-specific fecal IgA (Fig 6c). Mucosal MPER-specific humoral responses corresponded with SlpA-specific endpoint titers. *Nod+/+* mice had a significantly higher (approximately 1 log) endpoint titer for serum SlpA-specific IgG, vaginal IgA, and cecal IgA (Fig 6e). All animals were positive for SlpA-specific fecal IgA. MPER- and SlpA-specific humoral responses were additionally not correlated to histological alterations in spleen, colon, or small intestine (S2 Fig. Histological evaluation of large and small intestine in BALB/c mice.). Similar to the C57BL/6 model, the production of antigen-specific IFN-γ within BALB/c mouse tissues, as measured by ELISpot, was also not dependent on *Nod2* status (Fig 6f).

## Discussion

In this study we characterized the roles of TLR2, NOD2, and caspase-1 in macrophage phagocytosis, activation, and cytokine production in response to *L*. *gasseri* NCK1785, *L*. *acidophilus* NCFM, and the NCFM mutant strains NCK2025 and NCK2031. We conclude that NCK1785 and NCFM lead to the generation of a type II M2 (M2b)-like macrophage *in vitro*, a response dependent on expression of LTA and surface layer proteins in NCFM. We demonstrated the NOD2 stimulating capacity of MDP from lactobacilli strains NCK1785 and NCFM, adding to the previously described PRR stimulation profile, and found *Nod2-/-* macrophages were fundamentally impaired in their response to lactobacilli [16, 35]. Furthermore, robust mucosal and systemic antigen-specific humoral responses of mice mucosally immunized with a *L*. *acidophilus* NCFM-based vaccine expressing cell surface HIV-Gag or HIV-MPER was dependent on NOD2 signaling [6, 20].

*In vitro* macrophage differentiation leads to a polarized response classified as M1 or M2 (with additional M2a, M2b, and M2c subsets) depending on the specific activation cues delivered by vehicles such as lactobacilli strains [44, 45]. The M2b subset results from stimulation by TLRs, IL-1, and/or immune complexes and is characterized by high surface expression of MHCII along with increased production of anti-inflammatory IL-10 and pro-inflammatory IL-1, IL-6, and TNF-α in conjunction with diminished IL-12 [44, 46, 47]. Thus, NCK1785- and NCFM-stimulated macrophages exhibit M2b traits with co-expression of both pro- and anti-inflammatory mediators.

IL-10 and IL-12 (p70) are commonly evaluated as indicators of Th1 versus Th2/Treg outcomes in the context of lactobacilli immune stimulation [10]. Here, despite similar M2b traits, the cytokine profile associated with NCFM was biased toward higher IL-10 and IL-12 (p40), and lower KC than NCK1785. In agreement with previous reports, we show that NCFM LTA has an inflammatory effect since NCK2025, which lacks LTA, is associated with increased IL-10, decreased IL-12 (p40), and decreased CD11b and MHCII expression by macrophages *in vitro* [23, 24, 48]. Interestingly, TLR2 itself was required for maximal IL-10 production in response to lactobacilli, suggesting that the LTA-TLR2-IL10 axis is complicated by additional PRR ligands or perhaps TLR2 co-receptors [13, 49]. The SlpB/X deficient mutant, NCK2031, had an anti-inflammatory effect on BMDM, with decreased MHCII, IL-6, and MIP-1α compared to wild type NCFM. This is discrepant from previous reports of NCK2031 and is most likely due to the cell type evaluated and culture conditions employed [11, 24, 45]. The immunomodulatory properties of S-layer proteins from different lactobacilli species and strains must be further investigated.

Phagocytosis and lysosomal degradation of lactobacilli have previously been correlated with variable cytokine responses presumably due to the differential liberation of PRR-binding cell wall components [13, 27, 50]. We found differential susceptibility of NCK1785 and NCFM to lysozyme degradation, but this did not correlate with NOD2 activation or macrophage cytokine production. Phagocytosis and NOD2 signaling did correlate with maximal MHCII and CD11b co-expression for NCK1785 and NCFM. NOD2 activation was essential for cytokine responses and acted synergistically with TLR2 rather than as a negative regulator thereof [15, 51, 52].

The critical role of NOD2 for lactobacilli-induced innate immune signaling was further linked to adaptive immune responses using an *in vivo* mucosal vaccination model. Consistent with other reports, we observed that robust antigen-specific humoral responses against our NCFM-based mucosal vaccines required NOD2 signaling in C57BL/6 and BALB/c mice [51, 53–55]. No specific vaccine correlates were identified here, but the significant increase of gut IL-1β in NCK2166 immunized C57BL/6 mice was also dependent on NOD2. NOD2 and caspase-1 inflammasome activation were also required for lactobacilli induced IL-1β in BMDM. Caspase-1 inflammasome activation can result in adjuvant-associated, highly inflammatory pyroptosis; however, lactobacilli BMDM activation occurred in a pyroptosis-independent manner [56, 57]. While NOD2 is involved in both NALP1 and NALP3 inflammasome activation, future studies are required to define the constituents of the inflammasome in this setting and the implications of limited pyropotosis for vaccine adjuvancy [18, 58, 59].

A growing body of evidence suggests a crucial role for NOD2 and the microbiota in mucosal vaccine responses. Recent studies have demonstrated synergy between NOD2 and TLR2 in the development of antigen-specific humoral responses [51, 54]. While TLR2 has been shown to promote IgA class switching and plasma cell differentiation, Kim *et al* demonstrated the requirement of NOD2 for optimal humoral responses in a mucosal cholera toxin (CT) model [60, 61]. In their studies, the adjuvant effect of CT was promoted by microbiota-driven NOD2 signaling in CD11c^+^ phagocytes and implicated NOD2 in follicular helper T cells and plasma cell development [61]. Other studies have shown NOD2 expression by hematopoietic cells, in general, and B lymphocytes in particular, is required for optimal antibody responses [62–65]. NOD2 signaling by the microbiota appears necessary for the continuous diversification of memory B cells [65, 66]. NOD2 activation in the stromal compartment of the intestinal tract may also shape the antibody response [55]. Additional studies will be required to determine the specific cell types responsible for the NOD2-dependent humoral responses identified here.

Harnessing the power of the mucosal immune network for durable and anamnestic vaccination responses requires an understanding of the mucosal innate and adaptive immune systems [2]. Although more studies are required to understand the mechanism by which antigen-specific humoral responses are achieved by the mucosal NCFM vaccine platform, we have identified NOD2 as an essential regulating factor. Thus, with numerous tools available for the genetic manipulation of lactobacilli, these studies provide insight into pathways that can be exploited to enhance vaccine efficacy across model systems [26, 67, 68].

## Supporting information

S1 Table Persistence of NCK2166 in immunized mice at endpoint. (DOCX)

S1 Fig Histological evaluation of large and small intestine in C57BL/6 mice. *Nod2+/+* or *Nod2-/-* C57BL/6 mice were repeatedly immunized with STI buffer (buffer), NCK1895, or NCK2166. At sacrifice, large and small intestine were sampled, formalin fixed, paraffin embedded, and sections stained with hematoxylin and eosin in a routine fashion. Slides were evaluated by a board certified veterinary pathologist and characterized as normal, exhibiting mild colitis or enteritis (mild inflammation), or containing increased granulocytes in the lamina propria (proprial granulocytes). Vertical slice representation of (a) large intestine/colon and small intestine data shown as the proportion of animals per group in each classification. (b) Representative histological images of (1) normal colon, (2) mild colitis, (3) normal small intestine, (4) mild enteritis, and (5) increased proprial granulocytes. N = 4–6 mice per treatment group.
(TIF)

S2 Fig Histological evaluation of large and small intestine in BALB/c mice. *Nod2*+/+ or *Nod2-/-* BALB/c mice were repeatedly immunized with STI buffer (buffer), NCK1895, or GAD31. At sacrifice, large and small intestine were sampled, formalin fixed, paraffin embedded, and sections stained with hematoxylin and eosin in a routine fashion. Slides were evaluated by a board certified veterinary pathologist and characterized as normal, exhibiting mild colitis or enteritis (mild inflammation), or containing increased granulocytes in the lamina propria (proprial granulocytes). Vertical slice representation of (a) large intestine/colon and small intestine data shown as the proportion of animals per group in each classification. (b) Representative histological images of (1) normal colon, (2) mild colitis, (3) increased proprial granulocytes, (4) normal small intestine, and (5) mild enteritis. N = 11–12 mice per treatment group.
(TIF)

S1 Supporting materials and methods Preparation of AT-2 inactivated HIV IIIB, histology, and sample processing for lactobacilli enumeration. (DOCX)
