# Supplementary material for: Nod2 is required for antigen-specific humoral responses against antigens orally delivered using a recombinant Lactobacillus vaccine platform
Source: PLoS One. 2018 May 7;13(5):e0196950. doi: 10.1371/journal.pone.0196950 (PMC5937747; doi:10.1371/journal.pone.0196950)
Supplement: S1 Table — (DOCX) [file pone.0196950.s001.docx]

**S1 Table. Persistence of NCK2166 in immunized mice at endpoint.**

| **C57BL/6 mice** | **Treatment** | **Number of mice + for Em^R^ Bacteria** | |
| --- | --- | --- | --- |
|  |  | **MLN** | **IC** |
| ***Nod2+/+*** | Buffer | 0/6 | 0/6 |
|  | NCK1895 | 0/6 | 0/6 |
|  | NCK2166 | 1/6 | 3/6 |
| ***Nod2-/-*** | Buffer | 0/6 | 0/6 |
|  | NCK1895 | 0/6 | 0/6 |
|  | NCK2166 | 1/6 | 1/6 |

Wild type *Nod2+/+* or *Nod2-/-* C57BL/6 mice were repeatedly immunized with STI buffer (buffer), NCK1895, or NCK2166. At sacrifice, mesenteric lymph node (MLN) and ileal contents (IC) were collected, aseptically homogenized, and plated on erythromycin containing MRS agar. After overnight anaerobic incubation for erythromycin resistant (Em^R^) colony enumeration. Data is shown as the proportion of mice with positive cultures over the total number immunized per group.
