# Supplementary figures and images for: Nod2 is required for antigen-specific humoral responses against antigens orally delivered using a recombinant Lactobacillus vaccine platform
Source: PLoS One. 2018 May 7;13(5):e0196950. doi: 10.1371/journal.pone.0196950 (PMC5937747; doi:10.1371/journal.pone.0196950)

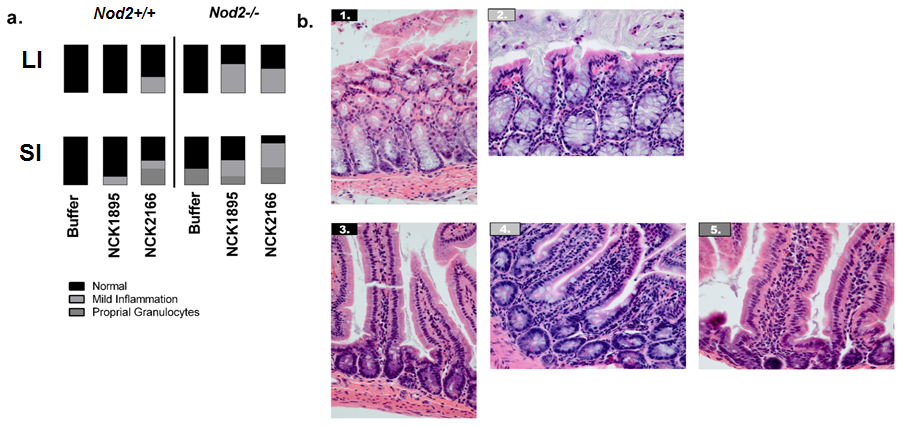

Supplement: S1 Fig — Nod2+/+ or Nod2-/- C57BL/6 mice were repeatedly immunized with STI buffer (buffer), NCK1895, or NCK2166. At sacrifice, large and small intestine were sampled, formalin fixed, paraffin embedded, and sections stained with hematoxylin and eosin in a routine fashion. Slides were evaluated by a board certified veterinary pathologist and characterized as normal, exhibiting mild colitis or enteritis (mild inflammation), or containing increased granulocytes in the lamina propria (proprial granulocytes). Vertical slice representation of (a) large intestine/colon and small intestine data shown as the proportion of animals per group in each classification. (b) Representative histological images of (1) normal colon, (2) mild colitis, (3) normal small intestine, (4) mild enteritis, and (5) increased proprial granulocytes. N = 4–6 mice per treatment group. (TIF) [file pone.0196950.s002.tif]

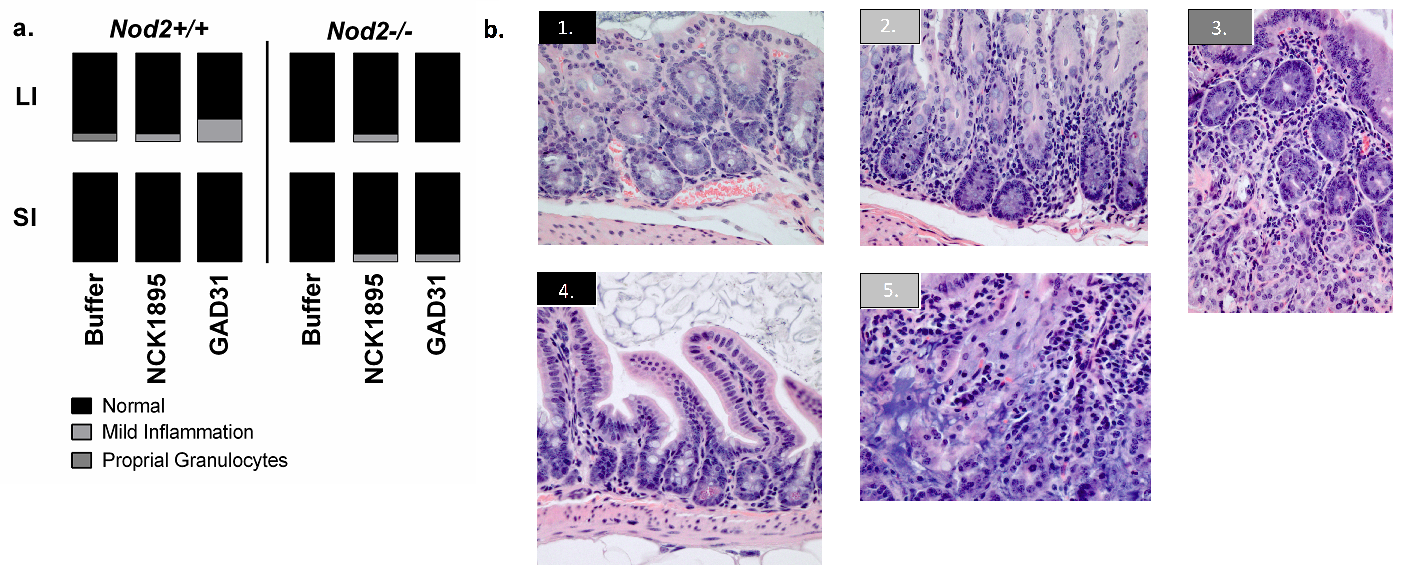

Supplement: S2 Fig — Nod2+/+ or Nod2-/- BALB/c mice were repeatedly immunized with STI buffer (buffer), NCK1895, or GAD31. At sacrifice, large and small intestine were sampled, formalin fixed, paraffin embedded, and sections stained with hematoxylin and eosin in a routine fashion. Slides were evaluated by a board certified veterinary pathologist and characterized as normal, exhibiting mild colitis or enteritis (mild inflammation), or containing increased granulocytes in the lamina propria (proprial granulocytes). Vertical slice representation of (a) large intestine/colon and small intestine data shown as the proportion of animals per group in each classification. (b) Representative histological images of (1) normal colon, (2) mild colitis, (3) increased proprial granulocytes, (4) normal small intestine, and (5) mild enteritis. N = 11–12 mice per treatment group. (TIF) [file pone.0196950.s003.tif]
