# Supplementary material for: Nod2 is required for antigen-specific humoral responses against antigens orally delivered using a recombinant Lactobacillus vaccine platform
Source: PLoS One. 2018 May 7;13(5):e0196950. doi: 10.1371/journal.pone.0196950 (PMC5937747; doi:10.1371/journal.pone.0196950)
Supplement: S1 Supporting materials and methods — (DOCX) [file pone.0196950.s004.docx]

**Supporting materials and methods**

**Preparation of AT-2 inactivated HIV IIIB**

Aldrithiol-2 (AT-2) inactivated HIV IIIB protein was prepared by BioMARC (CSU, Fort Collins, CO) for the purposes of this study following standard protocols. Briefly, 1 × 10^6^ A3.01 cells were obtained from the NIH AIDS Reagent Program (Rockville, MD) and grown under standard conditions in complete RPMI media (RPMI 1640 with L-Glutamine supplemented with 10% (v/v) FBS, 2 mM L-Glutamine, 10mM HEPES, 100 U/mL penicillin and 100 µg/mL Streptomycin) for 48 h. Cells were subsequently suspended in RPMI infection media (RPMI 1640 with L-Glutamine supplemented with 2.5% (v/v) FBS, 2mM L-Glutamine, and 10mM HEPES) along with HIV IIIB virus inoculum (H9/HTLV-III_B_ NIH 1983 supernatant; MOI 0.1 to 0.0001) and incubated for 1 h with intermittent swirling prior to seeding infected cells for HIV cultivation (1 × 10^6^ cells/T-150 cm^2^ flask) for a minimum of 10 days at 37 ˚C. Cultivated virions were harvested from the cellular supernatant via centrifugation (2,400 rpm for 10 min at 4 ˚C) and treated with AT-2 (250 µM) for 1 h at 37 ˚C prior to isolation via ultracentrifugation (1 h at 100,000 x g, 4 ˚C) with 15% glycerol underlay. Prior to usage in antigen-specific colorimetric ELISA assays, p24 protein quantification of stock was performed via HIV-1 p24 colorimetric ELISA (Xpress Bio, Frederick, MD) following manufacturer’s instructions and AT-2 inactivated virus was sonicated four times at 12 V for 15 second bursts on ice.

**Histology**

At sacrifice, samples of spleen, duodenum, jejunum, ileum, colon, and mesenteric lymph node were harvested and fixed in 10% formalin. Tissues were paraffin embedded, stained with standard hematoxylin and eosin, and evaluated by a board certified veterinary pathologist. Normal intestinal tissue (score = 1) was characterized by tightly packed crypts with up to 12 lymphocytes and/or plasma cells per villus tip in cross-section along with few granulocytes (no more than 7 per high power field) and no apparent crypt or surface injury. Mild enteritis/colitis (score = 3) was characterized by 12-18 lymphocytes and/or plasma cells per villus tip in cross-section or crypts separated by 4-5 cell layers of lymphocytes and/or plasma cells, with no crypt or surface injury apparent. Animals not classified as exhibiting mild enteritis/colitis,but having up to 10 granulocytes per high power field in the lamina propria were noted as having increased proprial granulocytes (score = 2).

**Sample processing for lactobacilli enumeration**

At sacrifice, mesenteric lymph nodes from immunized mice were excised, cleaned of residual fat, aseptically weighed in microcentrifuge tubes, and individually homogenized (Kimble Chase Kontes pellet pestle system, Vineland NJ). Additionally, ileal contents were collected into microcentrifuge tubes, suspended at 100 mg/mL in sterile PBS, homogenized (FastPrep-24), and supernatant collected post-centrifugation. Tissue homogenate and ileal content supernatant were plated on MRS agar (BD, Franklin Lakes, NJ) with 5 μg/mL erythromycin, and anaerobically incubated overnight for colony enumeration.
